# Huaier aqueous extract induces apoptosis of human fibrosarcoma HT1080 cells through the mitochondrial pathway

**DOI:** 10.3892/ol.2015.2906

**Published:** 2015-01-27

**Authors:** YANG CUI, HONGMEI MENG, WEIDONG LIU, HUAN WANG, QINGPENG LIU

**Affiliations:** 1 Department of Orthopedics, The Second Affiliated Hospital of Harbin Medical University, Harbin, Heilongjiang 150028, P.R. China; 2 Department of Orthopedics, The First Affiliated Hospital of Harbin Medical University, Harbin, Heilongjiang 150001, P.R. China; 3 Department of Orthopedics, The First Hospital of Qiqihar, Qiqihar, Heilongjiang 161000, P.R. China

**Keywords:** HT1080 cells, Huaier aqueous extract, apoptosis, mitochondrial pathway, cell motility

## Abstract

In recent years, aqueous extract of *Trametes robiniophila* Murr. (Huaier), a traditional Chinese medicine, has been frequently used in China for complementary cancer therapy. However, the mechanisms underlying its anticancer effects have yet to be elucidated. The present study aimed to evaluate the ability of Huaier extract to inhibit proliferation, promote apoptosis and suppress mobility in the fibrosarcoma HT1080 cell line *in vitro*. The cells were treated with gradient doses of Huaier extract at concentrations of 0, 4, 8 or 16 mg/ml for 24, 48 or 72 h. The cell viability and motility were measured *in vitro* using MTT, invasive, migration and scratch assays. The distribution of the cell cycle and the extent of cellular apoptosis were analyzed by flow cytometry. The apoptotic pathways were detected using a mitochondrial membrane potential transition assay and western blotting. The results revealed that the cellular viability decreased significantly with increasing concentrations of Huaier extract. In addition, cell invasiveness and migration were also suppressed significantly. It was demonstrated that Huaier extract induced G_2_ cell-cycle arrest and cellular apoptosis in a time- and dose-dependent manner. The decreased mitochondrial membrane potential, the downregulation of B-cell lymphoma 2 and pro-caspase-3, and upregulation of Bcl-2-associated X protein, cleaved caspase-9 and caspase-3 suggested that Huaier extract induced the apoptosis of HT1080 cells through the mitochondrial pathway. The results of the present study indicate that Huaier extract is a potential complementary agent for the treatment of fibrosarcoma.

## Introduction

Fibrosarcoma of the bone is a relatively rare soft-tissue tumor of mesenchymal origin that may occur throughout the body. Overall, it accounts for <5% of all bone sarcomas (1). Although morbidity resulting from fibrosarcoma is rare, patient survival rates are low. In recent years, long-term survival rates have remained poor despite advances in medical and surgical treatments. Fibrosarcoma of the bone is a highly fatal disease, which significantly affects patient survival.

At present, surgery with or without complementary therapies is the standard treatment for patients with fibrosarcoma of the bone. Although surgery is the accepted treatment for fibrosarcoma of the bone, adjuvant therapies, such as radiotherapy, chemotherapy and Traditional Chinese Medicine (TCM) may also be of potential clinical benefit. Of the complementary therapies, TCM has become of increasing interest in previous years, due to its antitumor effects and low toxicity (2–4).

Huaier aqueous extract, obtained from *Trametes robiniophila* Murr., is a type of officinal fungi that has been used in TCM for ~1600 years. However, the antitumor effects and underlying mechanism of Huaier extract have only been studied in recent decades. A previous study reported that the effective components of Huaier extract comprise 41.53% polysaccharides, 12.93% amino acids and 8.72% water (5). As a complementary antitumor agent, Huaier extract may promote cellular apoptosis, inhibit cellular proliferation and mobility, suppress angiogenesis, increase chemotherapy efficacy and strengthen systemic immune function (6–9). Previous studies have revealed that Huaier extract induces apoptosis in a number of tumor cells, including hepatocarcinoma, breast cancer and ovarian cancer cells (10–11). However, the effect of Huaier extract on the biological behavior of fibrosarcoma cells is yet to be elucidated. The present study aimed to demonstrate the antitumor effects of Huaier extract on the HT1080 cell line.

## Materials and methods

### Preparation of Huaier aqueous extract

The electuary Huaier ointment was provided by Qidong Gaitianli Medicine Co., Ltd. (Qidong, Jiangsu, China). In total, 2 g of the electuary ointment was dissolved in 20 ml complete medium and sterilized with 0.22 μm filter to obtain a 100 mg/ml stock solution, which was subsequently stored at −20°C.

### Cell culture

The fibrosarcoma HT1080 cell lines were obtained from the American Type Culture Collection (Manassas, VA, USA). The cells were cultured in Dulbecco’s modified Eagle’s medium and nutrient mixture F-12 (DMEM/F12) supplemented with 10% fetal bovine serum (FBS), 100 units/ml penicillin and 100 μg/ml streptomycin and stored at 37°C in a 5% CO_2_ atmosphere.

### Cell viability assay

The fibrosarcoma HT1080 cells were plated into 96-well plates. Subsequent to an overnight incubation, the cells were treated with gradient doses of Huaier extract (0, 4, 8 or 16 mg/ml) for 24, 48 or 72 h and then washed with phosphate-buffered saline (PBS). In total, 20 μl MTT dye (5 mg/ml) was added to each well, and the mixture was incubated for 4 h at 37°C. Following incubation, the cells were washed with PBS. Next, 150 μl dimethyl sulfoxide was added to each well to terminate the MTT reaction and dissolve the formazan crystals. Subsequent to agitation for 10 mins at room temperature, the optical density of the cells in each well was measured at 570 nm using a microplate reader (Bio-Rad 680; Bio-Rad Laboratories, Inc., Hercules, CA, USA). The percentage cell viability was calculated based on an untreated HT1080 control cell viability of 100%, as follows:

$$\text{Cell viability }(\%)=\frac{({\text{OD}}_{\text{treated well}}-{\text{OD}}_{\text{blank}})\times 100}{\left({\text{OD}}_{\text{untreated well}}-{\text{OD}}_{\text{blank}}\right)}$$

### Effect of Huaier on cell morphology

The HT1080 cells were incubated with Huaier extract at a concentration of 4 or 8 mg/ml for 24 or 48 h. In order to identify the morphological changes of the HT1080 cells, the cells were observed under an Olympus light microscope (CX31-72C02; Olympus Corporation, Tokoyo, Japan), and photomicrographs were captured using an Olympus digital camera (DP72; Olympus Corporation).

### Cell-cycle analysis

In total, 1×10^5^ cells/well were seeded into six-well plates and starved in serum-free medium at 37°C. Subsequent to a 12-h starvation period, the cells were treated with Huaier extract at concentrations of 0, 4 or 8 mg/ml for 24 or 48 h. The cells were then trypsinized and washed with PBS prior to being suspended with 500 μl RNase A (1 mg/ml) and 100 μg/ml propidium iodide (PI) (Beyotime Institute of Biotechnology, Haimen, Jiangsu, China) in order to stain the cellular DNA. Subsequent to a 20-min incubation period at room temperature in the dark, the DNA content of the cells was analyzed using a FACSCaliber flow cytometer (BD Biosciences, Franklin Lakes, NJ, USA) and the data was analyzed using ModFitLT software, version 2.0 (BD Biosciences).

### Flow cytometric analysis of cellular apoptosis

In total, 1×10^5^/well HT1080 cells were seeded into six-well plates and cultured overnight. Subsequent to treatment with Huaier extract at 4 or 8 mg/ml for 24 or 48 h, the cells were digested with 2.5 mg/ml trypsin, washed twice with PBS and then suspended with 300 μl binding buffer. Next, 2 μl Annexin V and 5 μl PI was added to the mixture. The cells were then incubated for 5–15 min at room temperature, and the ratio of early and late apoptotic cells was detected using a FACSCaliber flow cytometer.

### Mitochondrial membrane potential transition assay

In total, the HT1080 cells were seeded into 24-well plates at a density of 2×10^4^ cells/well. Subsequent to culturing with Huaier extract at a concentration of 8 mg/ml for 24 h, the cells were washed once with PBS. Next, 1 ml lipophilic cation 5,5′,6,6′-tetrachloro-1,1′,3,3′-tetraethylbenzimidazolcarbocyanine iodide (JC-1) staining medium (5 mg/ml in DMEM/F12 supplemented with 10% FBS) was added to each well, and the cells were cultured in the dark for 20 min at 37°C in a 5% CO_2_ atmosphere. The cells were then washed three times with JC-1 staining buffer and cultured in DMEM/F12 supplemented with 10% FBS. The mitochondrial membrane potential was detected using an Olympus confocal imaging system (Olympus FV100; Olympus Corporation).

### Western blot analysis

First, the cells were collected and the total cell lysates were prepared using lysis buffer (Beyotime Institute of Biotechnology). The protein concentration was then measured according to the bicinchoninic acid method, with bovine serum albumin (BSA) as the standard (12). Next, 20–40 μg equal amounts of whole cell lysates were loaded into 8–12% SDS-polyacrylamide gels for electrophoresis at constant voltage, prior to being transferred to a PVDF membrane (Millipore, Billerica, MA, USA) at a 200 mA constant current for 3 h. The membrane was then incubated in blocking buffer comprising 5% skim milk in Tris-buffered saline with Tween-20 (TBST) at room temperature for 2 h. Next, the blocked membrane was incubated with monoclonal mouse anti-human B-cell lymphoma 2 (Bcl-2; dilution, 1:500; cat. no. sc-7382), polyclonal rabbit anti-mouse Bcl-2 associated X protein (Bax; dilution, 1:500; cat. no. sc-6263), monoclonal mouse anti-human caspase-9 (dilution, 1:500; cat. no. sc-81663) and polyclonal rabbit anti-human caspase-3 (dilution, 1:500; cat. no. sc-7148) primary antibodies (Santa Cruz Biotechnology, Inc., Dallas, TX, USA) at 4°C overnight. The membrane was then washed with TBST three times every 10 min, and then incubated with a secondary goat anti-mouse or goat anti-rabbit (dilution, 1:1,000; cat. nos. sc-2031 and sc-2030, respectively; Santa Cruz Biotechnology, Inc.) at room temperature for 1 h. Subsequent to washing in TBST, immunoreactive bands were visualized using an enhanced chemiluminescence kit (Chemiluminescence Plus; Pierce Biotechnology, Inc., Rockford, IL, USA). The blots were also incubated with an polyclonal rabbit anti-human β-actin antibody (dilution, 1:1,000; cat. no. sc-130656; Santa Cruz Biotechnology, Inc.), which acted as an internal control for the quantity of target protein.

### Invasion assay

The invasive ability of the HT1080 cells was detected using the Transwell system, which consisted of 24 wells, an 8-μm pore size and a polycarbonate membrane coated with Matrigel (Corning Incorporated, NY, USA). First, the cells were starved for 12 h in serum-free medium. The cells were then digested using 0.25% trypsin. Next, the control and Huaier group cells were suspended in serum-free medium containing either 0.1% BSA or 0.1% BSA supplemented with 4 mg/ml Huaier extract, respectively. In total, 600 μl complete medium containing 5% FBS was added to each lower chamber, and 2×10^4^ cells in 100 μl serum-free medium containing 0.1% BSA with or without 4 mg/ml Huaier extract were added to each upper chamber. Subsequent to 17-h incubation, the cells attached to the upper surface of the membrane were removed with cotton swabs, and the cells adhered to the lower surface were stained with 1 mg/ml crystal violet. The total number of cells was counted in five representative high-power fields using an Olympus light microscope (CX31-72C02, Olympus Corporation).

### Migration assay

The migration of the HT1080 cells was detected using a Transwell system. However, in contrast to the invasion assay, the polycarbonate membrane of the lower chamber was not coated with Matrigel. In total, 600 μl complete medium with 5% FBS was added to each lower chamber, and 2×10^4^ cells in 100 μl serum-free medium containing 0.1% BSA with or without 4 mg/ml Huaier extract were added to each upper chamber. Subsequent to 12-h incubation at 37°C, the cells were stained and counted as described for the invasion assay.

### Scratch assay

In order to evaluate whether cellular migration was affected by the Huaier extract, a scratch assay of the HT1080 cells was performed. In total, 5×10^5^/well HT1080 cells were seeded into six-well plates. Reference points in close proximity to the scratch were marked to ensure an identical area of image acquisition. The cells were cultured overnight, scraped using a 10-μl pipette tip to create a straight-line scratch and then washed twice with PBS to remove any debris. Next, the cells were incubated with serum-free medium containing 4 mg/ml Huaier extract. Images of the scratch were then captured at 6 or 12 h and the images were quantitatively analyzed using ImageJ software. The distance between the edges of the scratch were measured and statistically analyzed.

### Statistical analysis

The data and results were confirmed following at least three independent experiments. The data are expressed as the mean ± standard deviation. An analysis of variance and Student’s t-test were used to determine any statistical significance. A value of P<0.05 was used to indicate a statistically significant difference.

## Results

### Huaier extract inhibits the viability and causes changes in the morphology of HT1080 cells

**Figure 1:**
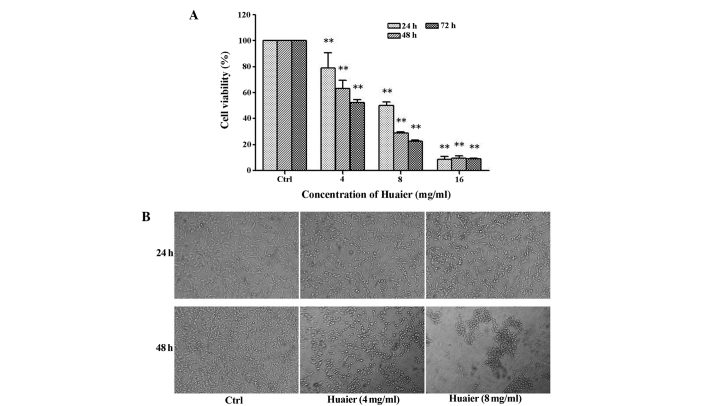
(A) Viability of HT1080 cells treated with gradient doses of Huaier for 24, 48 or 72 h, as determined by MTT assay. The viability of the HT1080 cells was inhibited in a dose- and time-dependent manner. (B) Phase-contrast images revealing the morphologies of HT1080 cells prior and subsequent to treatment with 0, 4 or 8 mg/ml Huaier for 24 or 48 h. The HT1080 cells treated with Huaier appeared smaller and apoptotic, and had detached from the walls of the culture flask. ^**^P<0.01 vs. control. Ctrl, control.

In order to determine the effect of Huaier extract on HT1080 cells, an MTT assay was performed to measure cellular viability. The cells were treated with Huaier extract at a concentration of 0, 2, 4, 8 or 16 mg/ml for 24, 48 or 72 h. As shown in Fig. 1A, the viability of the cells treated with Huaier extract was significantly lower compared with the control cells. Furthermore, it was revealed that Huaier extract significantly decreased the viability of HT1080 cells in a time- and dose-dependent manner.

The morphological changes of the HT1080 cells following treatment with Huaier extract at concentrations of 4 or 8 mg/ml for 24 or 48 h are shown in Fig. 1B. The HT1080 cells treated with Huaier extract had detached from the walls of the culture flask and appeared smaller and apoptotic, when compared with the untreated cells in the control groups. These morphological changes were representative of cellular damage following treatment with Huaier extract.

### Huaier extract induces cell cycle arrest and apoptosis in HT1080 cells

**Figure 2:**
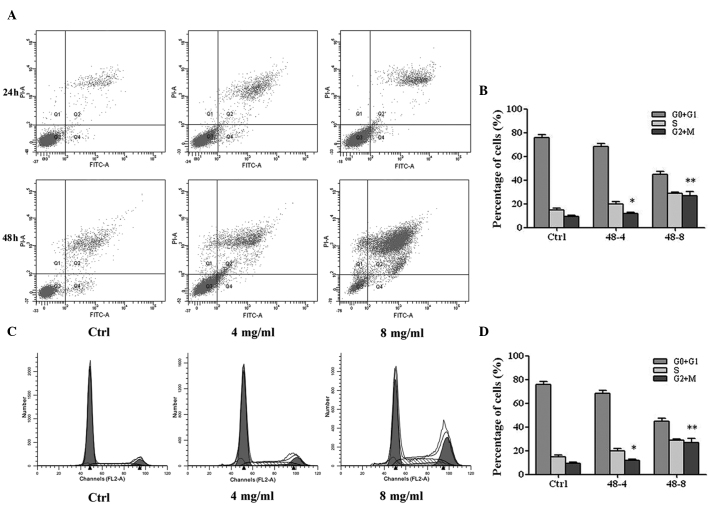
(A) The HT1080 cells were treated with Huaier at a concentration of 0, 4 or 8 mg/ml for 24 or 48 h. Flow cytometry with Annexin-V/PI was used to quantify Huaier-induced apoptosis in the HT1080 cells. (B) Flow cytometry revealed an increase in the proportion of apoptotic cells following treatment with increasing concentrations of Huaier. (C) HT1080 cells were treated with 4 or 8 mg/ml Huaier for 48 h. The cell cycle distribution of the HT1080 cells was assessed using flow cytometry following staining with PI. (D) Huaier significantly inhibited the proliferation of HT1080 cells in a dose-dependent manner via cell-cycle arrest at the G_2_ phase. ^**^P<0.01 vs. control. FITC, fluorescein isothiocyanate; PI, propidium iodide; Ctrl, control; A, area.

In the present study, Annexin V/PI staining and flow cytometry were used to analyze the rate of early apoptotic, late apoptotic and dead cells. The apoptotic rates of the HT1080 cells treated with 4 or 8 mg/ml Huaier extract for 24 or 48 h were significantly higher compared with the untreated control cells. As shown in Fig. 2A and B, Huaier extract leads to significant time- and dose-dependent increases in the rate of cellular apoptosis.

In addition, flow cytometry was also used to determine the ability of Huaier extract to induce cell cycle arrest or affect the cell cycle distribution of fibrosarcoma cells. The HT1080 cells were exposed to Huaier extract at a concentration of 4 or 8 mg/ml for 24 or 48 h. Compared with the control cells, the HT1080 cells treated with Huaier extract exhibited increased G_2_ arrest, caused by a decrease in the ratio of G_1_ and S phase cells and an increase in the ratio of G_2_ phase cells (Fig. 2C and D). These results revealed that Huaier extract suspends the proliferation of HT1080 cells via cell cycle arrest at the G_2_ phase.

### Mechanism behind the effect of Huaier extract on the apoptosis of HT1080 cells

**Figure 3:**
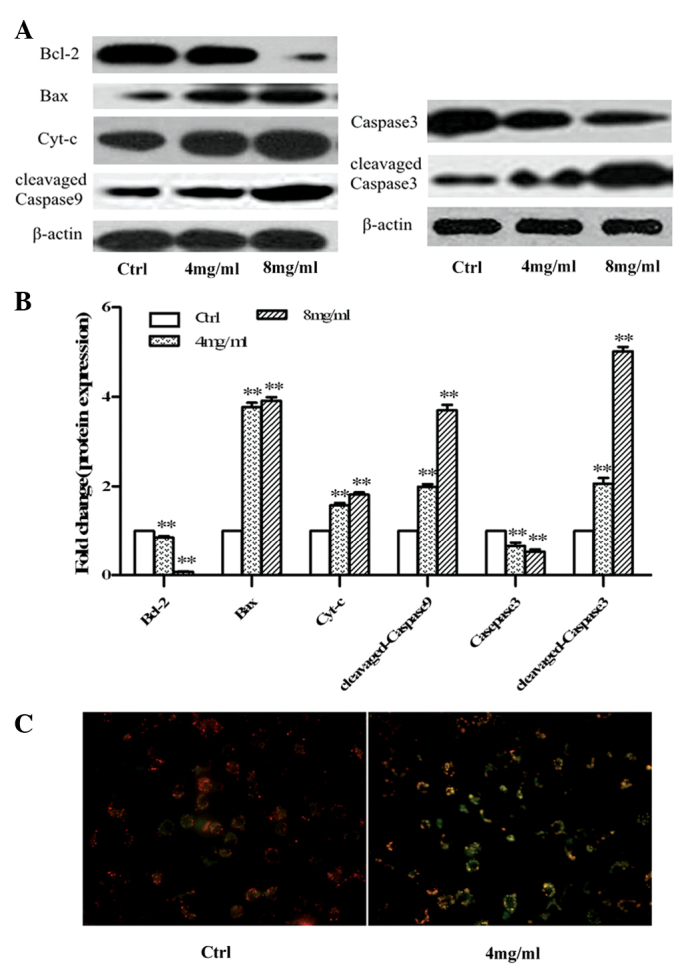
(A) HT1080 cells were treated with Huaier at concentrations of 0, 4 or 8 mg/ml for 48 h. The expression of B-cell lymphoma 2 (Bcl-2), Bcl-2 associated X protein (Bax), cytochrome-c (cyt-c), caspase-3 and caspase-9 was detected by western blotting. β-actin served as the internal control. (B) The relative expression of the target proteins in the HT1080 cells was analyzed. (C) HT1080 cells were treated with 4 mg/ml Huaier for 24 h. The green fluorescence was increased and the red fluorescence was decreased in the 4 mg/ml Huaier group compared with the control group. The mitochondrial membrane potential was detected using the JC-1 kit and an Olympus confocal imaging system.^**^P<0.01 vs. control.

In order to investigate the underlying mechanism of Huaier extract on cellular apoptosis, the expression of Bcl-2, Bax, caspase-9 and caspase-3 were analyzed using western blotting (Fig. 3A and B). In addition, the mitochondrial membrane potential transition was detected using the JC-1 assay and an Olympus confocal imaging system (Fig. 3C). In the Huaier-treated groups, a downregulation in the expression of Bcl-2 and pro-caspase-3, an upregulation in the expression of Bax and cleaved caspase-9 and-3 and a decrease in the mitochondrial membrane potential indicated that Huaier induced the apoptosis of fibrosarcoma cells through the mitochondrial pathway.

### Huaier extract suppresses the motility of HT1080 cells

**Figure 4:**
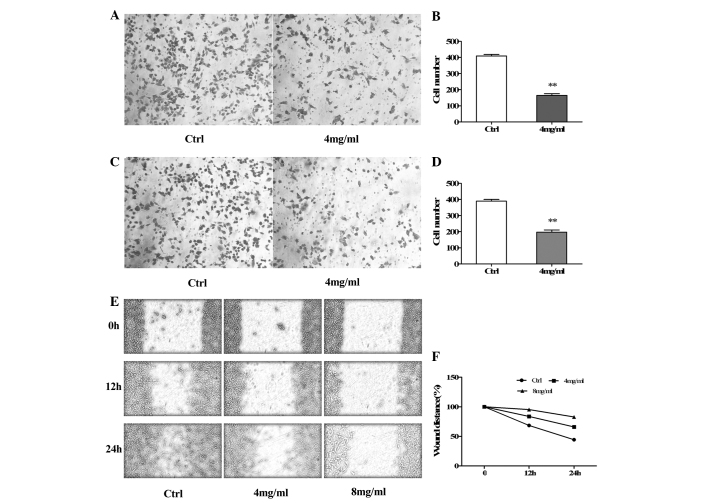
Cell mobility was strongly inhibited by Huaier extract in invasion and migration assays. (A) The invasive ability of the HT1080 cells following 17 h of treatment with Huaier was detected using the Transwell system. The cells were stained using the crystal violet staining agent. (B) The number of successfully invading HT1080 cells in the 4 mg/ml Huaier-treated group was significantly lower compared with the control group. (C) The migration ability of the HT1080 cells following 12 h of treatment with Huaier was detected using the Transwell system. The cells were stained using the crystal violet staining agent. (D) The number of successfully migrating HT1080 cells in the 4 mg/ml Huaier-treated group was significantly lower compared with the control group. (E) Images of the HT1080 cells treated with or without Huaier were captured at 0, 12 or 24 h during the scratch assay. (F) The distance between the edges of the scratch in the Huaier-treated group was greater than that of the control group. ^*^P<0.05 vs. control, ^**^P<0.01 vs. control.

The invasion, migration and scratch assays were performed in order to determine whether Huaier extract affected the motility of HT1080 cells *in vitro*. The results of the invasion assay revealed that the number of cells that had invaded through the Matrigel-coated polycarbonate membrane was significantly lower in the 4 mg/ml Huaier-treated groups than the control groups (Fig. 4A and B). These results indicated that Huaier extract suppressed the invasion ability of HT1080 cells.

Migration and scratch assays were performed in order to assess the migratory ability of the HT1080 cells treated with Huaier extract. Each of the assays revealed a consistent result. As shown in Fig. 4C and D, the number of cells in the 4 mg/ml Huaier-treated groups that had migrated through the polycarbonate membrane was significantly lower compared with the control groups. The migratory ability of the HT1080 cells was inhibited following treatment with Huaier extract.

The results of the scratch assay (Fig. 4A and B) revealed that the migration index, which corresponds to the wound healing capacity, was significantly decreased in the HT1080 cells treated with 4 or 8 mg/ml Huaier extract for 12 h compared with the untreated control cells. Similarly, the migration index was significantly decreased in HT1080 cells treated with 4 or 8 mg/ml Huaier extract for 24 h when compared with those of the control groups. The scratch assay revealed that Huaier extract suppressed the migratory ability of HT1080 cells.

## Discussion

Fibrosarcoma is a highly fatal disease that affects the survival of patients. A number of therapies, including radiotherapy and chemotherapy, are used to inhibit the growth of fibrosarcomas. These therapies are not only cytotoxic to tumor cells, but are also harmful to normal cells. Huaier, a TCM and complementary antitumor agent, has aroused interest due to its antitumor effects and low toxicity (2–4). In the present study, the antitumor effects and underlying mechanisms of Huaier extract in fibrosarcoma cells was investigated.

In the present study, fibrosarcoma HT1080 cells were treated with gradient doses of Huaier extract, at a concentration of 0, 4, 8 or 16 mg/ml. The effects of Huaier extract on the proliferation and apoptosis of HT1080 cells were observed and the antitumor mechanism was revealed. It was demonstrated that Huaier extract was able to inhibit cell viability and induce changes in cell morphology. The MTT assay revealed that Huaier extract inhibited the viability of HT1080 cells in a time- and dose-dependent manner. In addition, certain apoptotic morphological changes were observed following treatment with Huaier extract, which indicated cell damage. Due to the results of the MTT assay, the HT1080 cells were treated with Huaier extract at concentrations of 4 or 8 mg/ml in the subsequent experiments.

Zhang *et al* reported that Huaier extract induced cell cycle arrest at the G_2_ phase in the melanoma A875 cell line (13). The results from the present study revealed that the number of G_2_ phase HT1080 cells gradually increased with an increasing dose of Huaier extract, but the number of G_1_ and S phase cells decreased with an increasing concentration of Huaier extract. These results suggest that Huaier extract inhibits the proliferation of HT1080 cells by inducing cell cycle arrest at the G_2_ phase in a time and dose-dependent manner. This result is consistent with that of a previous study (13).

Previous studies have indicated that Huaier extract induces apoptosis in a number of cell types, including hepatocarcinoma, melanoma, ovarian cancer, cholangiocarcinoma and breast cancer cells (10,11,13–15). In the present study, the rate of apoptosis was analyzed using Annexin V/PI staining and flow cytometry. The results revealed that the rates of apoptosis in the Huaier-treated groups were significantly higher than those in the control groups. Furthermore, the rates of apoptosis gradually increased with increasing Huaier extract concentration and treatment time. Therefore, it can be concluded that Huaier extract induced the apoptosis of HT1080 cells in a dose- and time-dependent manner.

A decrease in mitochondrial membrane potential is a key event involved in the initiation of apoptosis, and one that is known to activate the apoptotic pathway (16–18). In the mitochondrial membrane potential transition assay, red fluorescence indicates a normal mitochondrial membrane potential, while an abnormal mitochondrial membrane potential exhibits green fluorescence. In the present study, cells of the Huaier-treated group demonstrated increased green fluorescence and decreased red fluorescence. This revealed that the mitochondrial membrane potentials of the Huaier-treated cells were lower compared with the control group cells. To further investigate the effect of Huaier extract on cellular apoptosis, the expression of a number of markers, namely Bax, Bcl-2, caspase-9 and caspase-3 was analyzed by western blotting. Bcl-2, an anti-apoptotic factor, is known to decrease the expression of the pro-apoptotic factor, Bax. Bax, a pro-apoptotic member of the Bcl-2 family, combines with Bcl-2 to form a dipolymer, which ultimately inactivates the Bcl-2 gene. This inactivation decreases the expression of pro-caspase-3 and increases the expression of cleaved caspase-3, which subsequently promotes cellular apoptosis (19–20). In the present study, the expression of Bcl-2 and pro-caspase-3 was significantly lower in the Huaier-treated groups compared with the control groups. By contrast, the expression of Bax and cleaved caspase-3 was significantly higher compared with the control groups. Caspase-9 is an apoptotic marker involved in the mitochondrial pathway (21). In the present study, the expression of cleaved caspase-9 was higher in the Huaier groups compared with the control groups. These findings revealed that Huaier induced apoptosis in HT1080 cells through the mitochondrial pathway.

In addition to investigating the effect of Huaier on the proliferation and apoptosis of fibrosarcoma HT1080 cells, the present study also determined the invasive and migratory ability of HT1080 cells treated with Huaier extract using the Transwell system. The invasion and migration assays revealed that the number of cells that passed through the polycarbonate membrane was significantly lower in the Huaier-treated groups compared with the control groups. This indicated that the invasive and migration capability of HT1080 cells significantly decreased following treatment with Huaier extract. The results of the scratch assay revealed that the migration index, which corresponds to the wound healing capacity, was significantly lower in the HT1080 cells treated with Huaier extract compared with the control groups. The scratch assay also revealed that Huaier extract suppressed the migration ability of HT1080 cells. This was concordant with the results of the migration assay in the Transwell system. This finding indicates that Huaier extract inhibited the mobility of HT1080 cells, a result that is in agreement with those of previous studies (5,11).

In summary, Huaier extract not only inhibited cellular proliferation by inducing apoptosis via the mitochondrial pathway, but also induced cell cycle arrest and reduced the mobility of HT1080 cells. In order to identify additional mechanisms that underlie the action of Huaier on HT1080 cells, future studies are required.
